# A Computational Study of the Role of Secondary Metabolites for Mitigation of Acid Soil Stress in Cereals Using Dehydroascorbate and Mono-Dehydroascorbate Reductases

**DOI:** 10.3390/antiox11030458

**Published:** 2022-02-25

**Authors:** Shuvasish Choudhury, Muhammed Khairujjaman Mazumder, Debojyoti Moulick, Parul Sharma, Sandeep Kumar Tata, Dibakar Ghosh, Hayssam M. Ali, Manzer H. Siddiqui, Marian Brestic, Milan Skalicky, Akbar Hossain

**Affiliations:** 1 Plant Stress Biology and Metabolomics Laboratory, Department of Life Science and Bioinformatics, Assam University, Silchar 788011, India; 2 Central Instrumentation Laboratory, Assam University, Silchar 788011, India; khairujjaman1987@gmail.com (M.K.M.); drubha31@gmail.com (D.M.); 3 Department of Zoology, Dhemaji College, Dhemaji 787057, India; 4 Don Bosco School, Silchar 788003, India; parulsharmabotany@gmail.com; 5 Department of Botany, Balmiki Rajniti Mahila College, Munger University, Munger 811201, India; sandeeptata83@gmail.com; 6 Division of Agronomy, ICAR-Indian Institute of Water Management, Bhubaneswar 751023, India; dibakar.ghosh@icar.gov.in; 7 Department of Botany and Microbiology, College of Science, King Saud University, Riyadh 11451, Saudi Arabia; hayhassan@ksu.edu.sa (H.M.A.); mhsiddiqui@ksu.edu.sa (M.H.S.); 8 Department of Plant Physiology, Slovak University of Agriculture, Nitra, Tr. A. Hlinku 2, 94901 Nitra, Slovakia; marian.brestic@uniag.sk; 9 Department of Botany and Plant Physiology, Faculty of Agrobiology, Food, and Natural Resources, Czech University of Life Sciences Prague, Kamycka 129, 16500 Prague, Czech Republic; skalicky@af.czu.cz; 10 Department of Agronomy, Bangladesh Wheat and Maize Research Institute, Dinajpur 5200, Bangladesh

**Keywords:** AsA-GSH cycle, aluminum, cereal crops, DHAR, electrostatic interactions, manganese, MDHAR

## Abstract

The present study investigates the potential ameliorative role of seven secondary metabolites, viz., ascorbate (AsA), reduced glutathione (GSH), jasmonic acid (JA), salicylic acid (SA), serotonin (5-HT), indole–3–acetic acid (IAA) and gibberellic acid (GA3), for mitigation of aluminium (Al^3+^) and manganese (Mn^2+^) stress associated with acidic soils in rice, maize and wheat. The dehydroascorbate reductase (DHAR) and mono-dehydroascorbate reductase (MDHAR) of the cereals were used as model targets, and the analysis was performed using computational tools. Molecular docking approach was employed to evaluate the interaction of these ions (Al^3+^ and Mn^2+^) and the metabolites at the active sites of the two target enzymes. The results indicate that the ions potentially interact with the active sites of these enzymes and conceivably influence the AsA–GSH cycle. The metabolites showed strong interactions at the active sites of the enzymes. When the electrostatic surfaces of the metabolites and the ions were generated, it revealed that the surfaces overlap in the case of DHAR of rice and wheat, and MDHAR of rice. Thus, it was hypothesized that the metabolites may prevent the interaction of ions with the enzymes. This is an interesting approach to decipher the mechanism of action of secondary metabolites against the metal or metalloid - induced stress responses in cereals by aiming at specific targets. The findings of the present study are reasonably significant and may be the beginning of an interesting and useful approach towards comprehending the role of secondary metabolites for stress amelioration and mitigation in cereals grown under acidic soil conditions.

## 1. Introduction

Soil acidity is one of the major constraints that considerably affects global agricultural productivity. More than 40% of global arable lands are considered acidic, and they usually contain exceedingly high concentrations of aluminium (Al^3+^) and manganese (Mn^2+^), along with several other associated factors [1,2,3,4,5,6]. The presence of both Al^3+^ and Mn^2+^ at remarkably high concentrations remains as a major cause for the decline in crop yield and productivity in acidic soils [7]. While in neutral and basic soil conditions, aluminium exists as non-toxic forms such as aluminium oxide or aluminosilicates; however, it mobilizes to toxic trivalent cations (Al^3+^) under acidic soil conditions with pH < 5 [6,8]. The primary site of Al^3+^ injury is the plant root system, causing root growth inhibition [1,9,10,11,12,13]. Al^3+^ induces the production of reactive oxygen species (ROS) such as hydrogen peroxide (H_2_O_2_) and superoxide radical (O_2_^∙^^−^), and imparts oxidative stress, leading to loss of plasma membrane integrity and lipid peroxidation [14,15,16,17,18,19,20,21,22]. Besides causing damage to plasma membrane, Al^3+^–induced ROS production also results in growth inhibition, ATP depletion, inhibition of cellular respiration, alteration of redox homeostasis, mitochondrial dysfunction and metabolic alterations [22,23]. Like other stressors (abiotic) and metal/metalloid, Al^3+^-induced ROS production and subsequent alteration of physiological and cellular functions have been well documented in a large variety of plant species [24,25,26,27,28,29,30,31,32,33,34,35,36,37,38,39]. Manganese (Mn^2+^) toxicity is recognised as a major factor affecting plant growth and metabolism in acidic and poorly drained soils [40]. Besides its toxic impacts on plants at higher concentrations, Mn^2+^ is also a vital element required by plants for various physiological and cellular functions [41]. Mn^2+^ plays a crucial role during photosynthesis and also acts as co-factor for many important enzymes [42,43,44]. Some recent studies have demonstrated that the presence of Mn^2+^ at higher concentrations causes a strong detrimental impact on a plant’s physiological and biochemical functions, leading to cell death [44,45,46,47]. However, in comparison to plant responses to Al^3+^ in acidic soils, relatively very little is known regarding the basis of Mn^2+^ in plants.

Monodehydroascorbate reductase (MDHAR) and dehydroascorbate reductase (DHAR) are two major enzymes of the ascorbate–glutathione (AsA–GSH) cycle [48,49,50]. The AsA–GSH cycle is responsible for the detoxification of H_2_O_2_, produced through light-dependant photosynthetic reactions. During the ascorbate peroxidase enzyme mediated reduction of H_2_O_2_ to H_2_O, monodehydroascorbate (MDHA) radical is formed, which is then reduced to ascorbate by MDHAR or is unequally converted to ascorbate and dehydroascorbate (DHA) [48,49]. The DHA, thus formed, is converted to ascorbate by DHAR with the expense of reduced glutathione (GSH) to form oxidized glutathione (GSSG). The roles of plant hormones and secondary metabolites in regulating abiotic stress by modulating the antioxidant pool have been widely studied [51,52,53]. These include a diverse class of chemical compounds such as gibberellins (GA3), salicylic acid (SA), jasmonic acid (JA), serotonin (5-HT), ascorbic acid (AsA), glutathione (GSH), etc. The SA (or ortho hydroxyl benzoic acid) is a phenolic compound that regulates diverse aspects of plant responses to abiotic stresses [39,54,55,56]. SA can modulate stress responses and enhances stress tolerance by regulating the antioxidative defence system to scavenge the ROS production [57]. The role of SA in alleviating heavy metal and metalloid-induced stress responses have been reported in rice, maize, legumes and *Cassia tora* [54,58,59,60]. JA plays important role in regulating various essential processes in plants and is also believed to have a major ameliorating role in controlling the deleterious effects of various abiotic stresses [61,62]. Auxins or indole-3-acetic acid (IAA) and GA3 are important plant growth regulators which also play a major role in plants during abiotic stresses [63]. AsA and GSH are well known to ameliorate abiotic stresses by scavenging the toxic ROS produced in cells [64,65]. They are known to function stringently in controlling the levels of ROS produced during a wide range of stresses such as drought, salinity, heavy metals, metalloids, etc. [66,67,68]. Another metabolite that emerged as an important component in regulating abiotic stress in plants is serotonin (5-hydroxytryptamine, 5-HT) [52,69,70]. However, its precise role in regulating abiotic stress including Al^3+^ and Mn^2+^ is still not clearly known.

Growth regulators and metabolites are major components of plants which influence physiological and cellular functions. Though the signalling roles of these chemical entities during abiotic stress have been largely investigated, their specific functional role during abiotic stresses is largely unknown. Computational approaches to study the interaction of ions with antioxidant enzymes and the possible role of plant secondary metabolites thereon are seldom performed [28,29,59]. In this investigation, we studied the role of some plant growth regulators and secondary metabolites in ameliorating the possible Al^3+^ and Mn^2+^-induced stress in cereal crops by targeting MDHAR and DHAR as model antioxidant enzymes using an in silico approach.

## 2. Materials and Methods

### 2.1. The Target Enzymes

The three-dimensional structure of the DHAR of rice (*Os*DHAR) in complex with AsA was available at the RCSB Protein Data Bank (PDB) with id 5D9W. The structure was determined by X-ray diffraction at a resolution of 1.69 Å. Likewise, the structure of MDHAR from rice (*Os*MDHAR) with PDB id 5JCI, determined at 1.70 Å resolution employing X-ray diffraction, was available in PDB. These structures were downloaded from the database in *.pdb* format. The three-dimensional structures of DHAR and MDHAR of maize and wheat were obtained from the SWISS-MODEL Repository database (https://swissmodel.expasy.org/repository/. accessed on 20 June 2020 ), which is a repository of three-dimensional structures which were modelled using the SWISS-MODEL homology modelling tool [71]. The models of DHAR from wheat (*Ta*DHAR) and maize (*Zm*DHAR) with accession numbers Q84UH6 and B4FT31, respectively were obtained from this database. Models of MDHAR from maize (*Zm*MDHAR) and wheat (*Ta*MDHAR), bearing accession numbers C4J4E4 and K4HRS2, respectively, were obtained accordingly. All the structures were downloaded in *.pdb* format.

### 2.2. Ligands (Metabolites and Ions)

The two-dimensional structures of Al^3+^ and Mn^2+^ were obtained from the NCBI PubChem compounds database (https://pubchem.ncbi.nlm.nih.gov/, accessed on 20 June 2020), while three-dimensional structures of AsA, JA, SA, GSH, IAA, GA3, 5-HT, MDHA and DHA were downloaded as *.sdf* files from this database. The three-dimensional structure of MDHA was not available at the database, and hence the two-dimensional structure was downloaded and used as input file at the https://cactus.nci.nih.gov/translate/ (accessed on 20 June 2020). The output three-dimensional structure was downloaded as an .*sdf* file. The physical and chemical properties of the metabolites, such as molecular weight, molecular formula, topological polar surface area (TPSA), octanol/water partition coefficient (XlogP3) and numbers of hydrogen bond donor (HBD) and acceptor (HBA) groups were obtained from the PubChem compounds database. Details of the ligands are given in Table 1. The structures of DHA and MDHA were used as reference ligands, while others were test ligands.

### 2.3. Molecular Docking

Molecular docking is a powerful in silico tool which determines the interactions between a protein and a compound (called ligand). The strength of their interactions, site of interaction and optimal binding orientations are also determined during docking. In the present study, molecular docking was performed using Molegro Virtual Docker 6.0 (MVD), following [72,73]. MVD is a potent docking tool which provides over 87% accuracy [74]. For *Os*DHAR, the docking was performed at the AsA binding site, having coordinates X: 2.47; Y: −5.84; Z: 6.72, and amino acids within a radius of 20 Å were included in the docking site. The three-dimensional structure of *Zm*DHAR was obtained from SWISS-MODEL database. Available cavities of the structure were determined using MVD, and docking was accordingly performed at the cavities cantered around X: 0.50; Y: 31.04 and Z: −0.75, incorporating amino acid residues within a radius of 30 Å. *Ta*DHAR was docked at X: 11.06; Y: −0.51; Z: 9.75, incorporating amino acids within a radius of 20 Å. *Os*MDHAR was docked at the FAD-binding active site, with coordinates X; 28.94; Y: 7.29; Z: 17.67, including amino acids within a radius of 20 Å. *Zm*MDHAR was docked at X: −11.73; Y: 24.87; Z: −15.20, including amino acids within a radius of 30 Å. *Ta*MDHAR was docked at X: 27.13; Y: 17.74; Z: 15.78, including amino acids within a radius 25 Å.

The docking parameters used in the study include the MolDock scoring function, with 10 runs each of 1500 iterations for each ligand, and five best poses were retained, in terms of MolDoc score (i.e., highest free energy of binding of the ligand and the receptor), and they were selected for further analysis. For Al^3+^ and Mn^2+^, the five best poses were used for the analysis. Furthermore, the Rerank score and hydrogen bond score for each ligand with each target were recorded.

### 2.4. Visualization of the Interactions

The docking poses obtained following the docking using MVD were imported into the workspace to analyse the binding orientations and interactions. The electrostatic surfaces of the docked ligands or poses at the active site were generated for each ligand. The electrostatic surfaces of the best pose of all the docked secondary metabolites and the ions at the active site of the enzymes were individually developed. Following this, the surfaces were observed for possible overlap for each ion and metabolite to see if the surfaces of ions overlap with those of the secondary metabolites. Further, the amino acid residues of the active site of the receptors involved in different types of interactions (stearic, electrostatic and hydrogen bonding) with different ligands were visualised. In addition, energy maps were generated for each receptor/target to see the sites which favour these interactions and the ligands which interact at these sites.

### 2.5. Statistical Analysis

To identify the properties of the ligands which affect the docking scores, a correlation analysis was performed by determining Pearson’s correlation coefficient. The physical and chemical properties of the ligands considered for the analysis include molecular weight, TPSA, XlogP3, HBD and HBA groups present in the ligands, following Mazumder et al. [72,73]. These properties were analysed against MolDoc, Re-rank and Hydrogen bond scores. However, because docking scores are negative values, and smaller values represent better inhibition, all the docking scores were converted to positive values before the statistical analysis, following Choudhury et al. [59].

## 3. Results

### 3.1. Docking Scores against DHAR

All the ligands showed negative docking scores with the DHAR targets in rice, wheat and maize (Table 2). Against *Os*DHAR, JA shows the best MolDock score, while GSH showed the highest scores against *Zm*DHAR and *Ta*DHAR. Compared to Al^3+^, these docking scores are 3.61-fold, 2.51-fold and 4.38-fold higher for these metabolites with the targets. Similarly, the scores of these metabolites with the three targets are higher by 3.46-fold, 2.51-fold and 4.12-fold, respectively, compared to Mn^2+^. However, AsA showed the highest hydrogen bonding scores against all the targets.

### 3.2. Docking Scores against MDHAR

Against MDHAR of rice, wheat and maize, GSH showed the best docking (MolDock) scores, followed by GA3 (Table 3). The docking scores of GSH were found to be higher than the scores of Al^3+^ and Mn^2+^ by 3.07-fold, 3.17-fold and 4.36-fold respectively, against the MDHAR of rice, maize and wheat. Against all the three targets, GSH also shows the highest hydrogen bond scores, indicating that hydrogen bonding is critical in the interactions and docking scores.

### 3.3. Docking Orientation and Overlapping Surfaces

**Figure 1:**
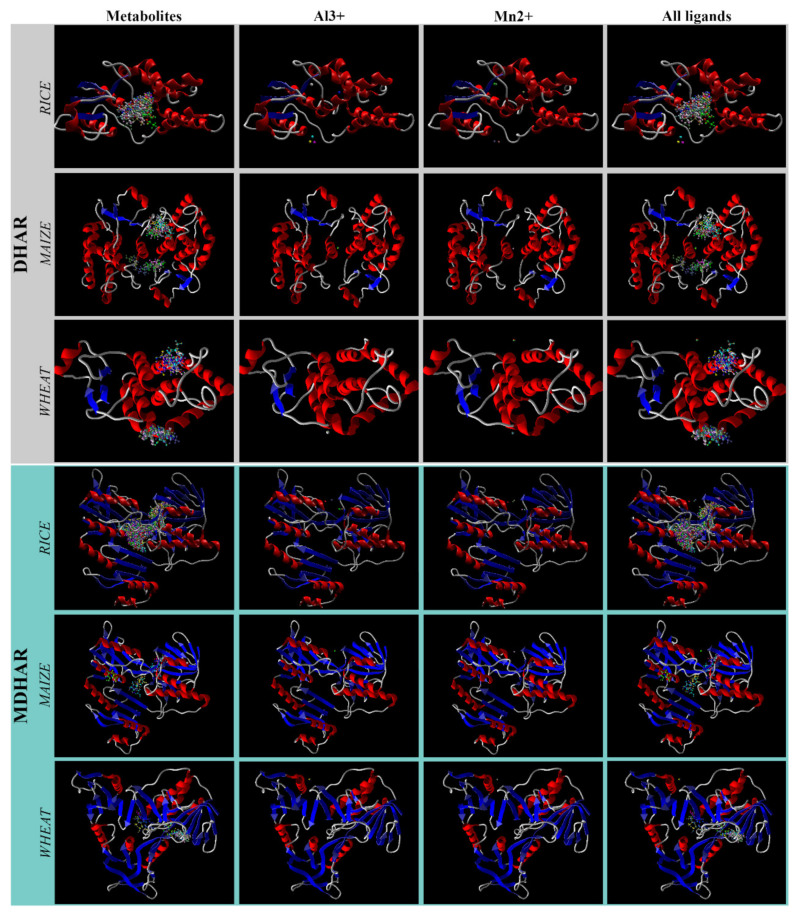
Docking poses of the metabolites and ions at the active sites of the six targets. The protein chain is shown in the secondary structures.

Against *Os*DHAR, the ions bind at three sites of the enzyme, while all the metabolites bind to a single active site. On the other hand, in the case of *Zm*DHAR, all the ions bind to the same site while the metabolites bind at two pockets, and there is no common binding pocket. Interestingly, against *Ta*DHAR, the metabolites bind to two pockets, Al^3+^ binds to a single site and Mn^2+^ binds to two sites. One of the sites of the binding of Mn^2+^ falls within one of the binding sites of the metabolites, while the other site and the site of binding of Al^3+^ are different from those of metabolites (Figure 1).

**Figure 2:**
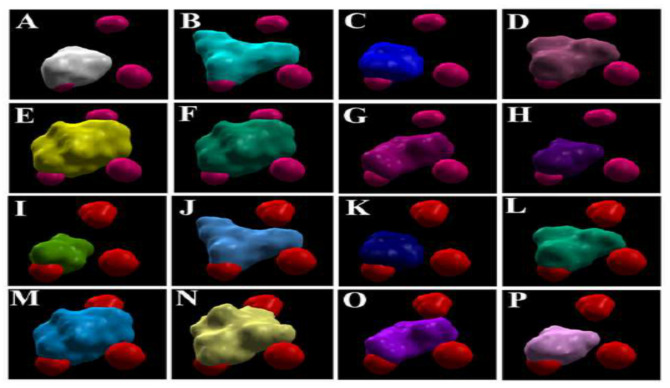
Overlapping electrostatic surfaces of metabolites and metals at the active site of *Os*DHAR. (**A**) AsA; (**B**) JA; (**C**) SA; (**D**) 5-HT; (**E**) GA3; (**F**) GSH; (**G**) IAA and (**H**) DHA with Mn^2+^; and (**I**) AsA; (**J**) JA; (**K**) SA; (**L**) 5-HT; (**M**) GA3; (**N**) GSH; (**O**) IAA and (**P**) DHA with Al^3+^. The spherical purple and red spheres represent the surfaces of Al^3+^ and Mn^2+^ ions, respectively.

**Figure 3:**
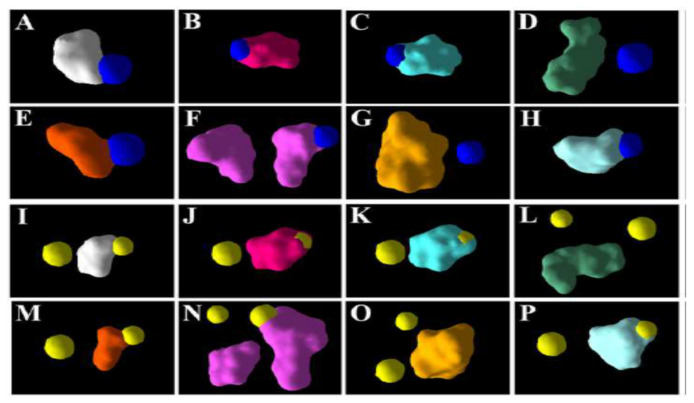
Electrostatic surfaces of the ions and metabolites and their overlaps in the case of *Ta*DHAR: (**A**) MDHA; (**B**) DHA; (**C**) AsA; (**D**) GSH; (**E**) SA; (**F**) 5-HT; (**G**) GA3; (**H**) IAA with Al^3+^. (**I**) MDHA; (**J**) DHA; (**K**) AsA; (**L**) GSH; (**M**) SA; (**N**) 5-HT; (**O**) GA3; (**P**) IAA with Mn^2+^. The dark blue and yellow spheres represent the electrostatic surfaces of Al^3+^ and Mn^2+^.

On developing electrostatic surfaces of the individual metabolites and the five best poses of the ions on *Os*DHAR, it was found that only one of the three poses of the ions partially overlap with each of the surfaces of the metabolites (Figure 2). In the case of *Zm*DHAR, there is no such overlapping of the surfaces (Figure S1). In the case of *Ta*DHAR, there is partial overlap of the electrostatic surfaces of MDHA, DHA, AsA, SA, 5-HT and IAA with that of Al^3+^, and as also revealed from the docking poses, there is overlap of the surfaces of one of the poses of Mn^2+^ with MDHA, DHA, AA, SA, 5-HT and IAA. Here, the surfaces of GSH and GA3 do not overlap with those of the ions (Figure 3).

**Figure 4:**
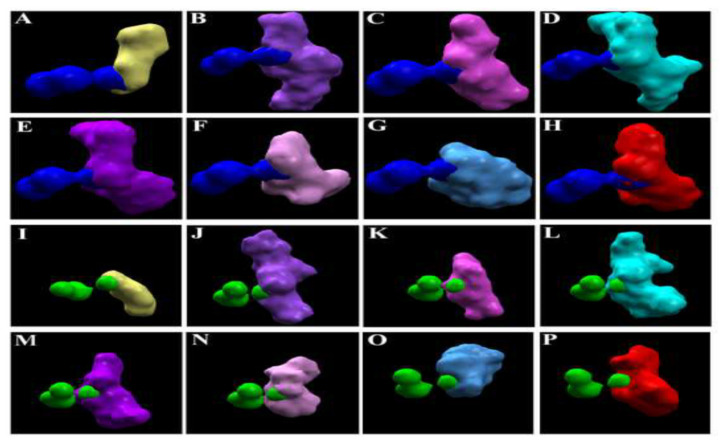
Electrostatic surfaces of the ligands at the active site of *Os*MDHAR. Overlapping electrostatic surfaces of docked ligands and metal ions. (**A**) MDHA; (**B**) GSH; (**C**) AsA; (**D**) JA; (**E**) 5-HT; (**F**) SA; (**G**) GA3 and (**H**) IAA with Al^3+^; and (**I**) MDHA; (**J**) GSH; (**K**) AsA; (**L**) JA; (**M**) 5-HT; (**N**) SA; (**O**) GA3 and (**P**) IAA with Mn^2+^. The dark blue spheres represent the surfaces of Al^3+^ and the green spheres represent Mn^2+^.

In *Os*MDHAR, the ions (Al^3+^ and Mn^2+^) bind to the same pocket of the enzyme as that of the metabolites with three different poses of each ion. However, out of the three poses of the two ions, the surfaces of only one of the poses overlaps with the surfaces of the metabolites (Figure 4). In the case of *Zm*MDHAR, the metabolites bind to three different pockets and the ions bind to an isolated single pocket. Thus, there is no overlap in the electrostatic surfaces (Figure S1). In the case of *Ta*MDHAR, all the metabolites bind to a single elongated pocket of the enzyme, and the ions bind to another isolated single pocket, and thus no overlap in the electrostatic surfaces is seen (Figure S1).

### 3.4. Energy Map

**Figure 5:**
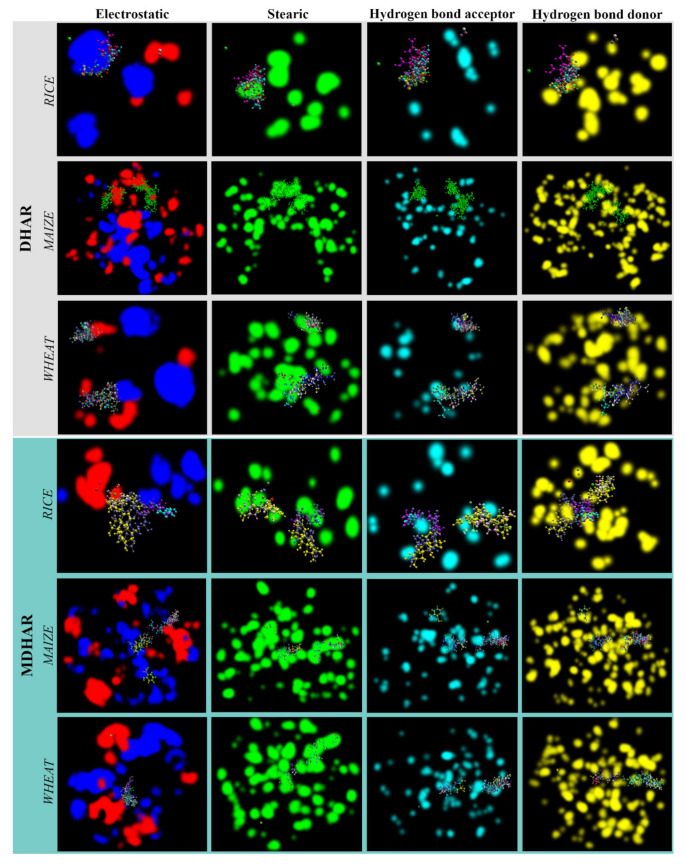
Energy maps of the receptors showing favourable surfaces for different types of interactions. The docked poses of the ligands including metabolites and ions are also shown.

With *Os*DHAR and *Zm*DHAR, the metabolites interact mainly in the electrostatic, stearic and HBD favourable regions, and not in the HBA region. However, the ions fall in the electrostatic and stearic favourable regions. In the case of *Ta*DHAR, the metabolites dock similarly at electrostatic, stearic, HBA and HBD interaction favourable regions, while the ions interact at electrostatic interaction favourable regions. The metabolites interact with all the favourable regions in the case of *Os*MDHAR, while the ions dock at the electrostatic and stearic interaction favourable regions. In the case of *Zm*MDHAR and *Ta*MDHAR, the metabolites interact more at the stearic favourable regions compared to other regions (Figure 5).

### 3.5. Ligand Properties Affecting the Interactions

Against *Os*DHAR, MolDock and Rerank scores are positively correlated to molecular weight, while hydrogen bond scores were found to be positively correlated to TPSA, HBD and HBA, and negatively correlated to XlogP3, albeit weakly. In the case of *Zm*DHAR, while the MolDock score has no correlation with any of the ligand properties studies, the hydrogen bond score was found to be negatively correlated to XlogP3, and the Rerank score was positively correlated to molecular weight, HBD and TPSA. Again, with *Ta*DHAR, both MolDock and Rerank scores were not correlated to any of the properties of the ligands, while the hydrogen bond score was found to be positively correlated to HBA and TPSA, and negatively correlated to XlogP3 (Table 4).

Thus, the results indicate that the hydrogen bond scores were negatively correlated to XlogP3 for all the targets, while TPSA and HBA are positively correlated to the hydrogen bond scores for *Os*DHAR and *Ta*DHAR. In case of all the MDHAR targets, MolDock scores were found to be mildly positively correlated to molecular weights, and the Rerank score was positively correlated only to the molecular weight in the case of *Ta*MDHAR. Interestingly, with the hydrogen bonding scores of all the targets, while XlogP3 was negatively correlated, HBD, HBA and TPSA were found to be positively correlated. The correlations, both positive and negative, in the case of the hydrogen bond scores were stronger (Table 5).

### 3.6. Interactions of Different Metabolites

**Figure 6:**
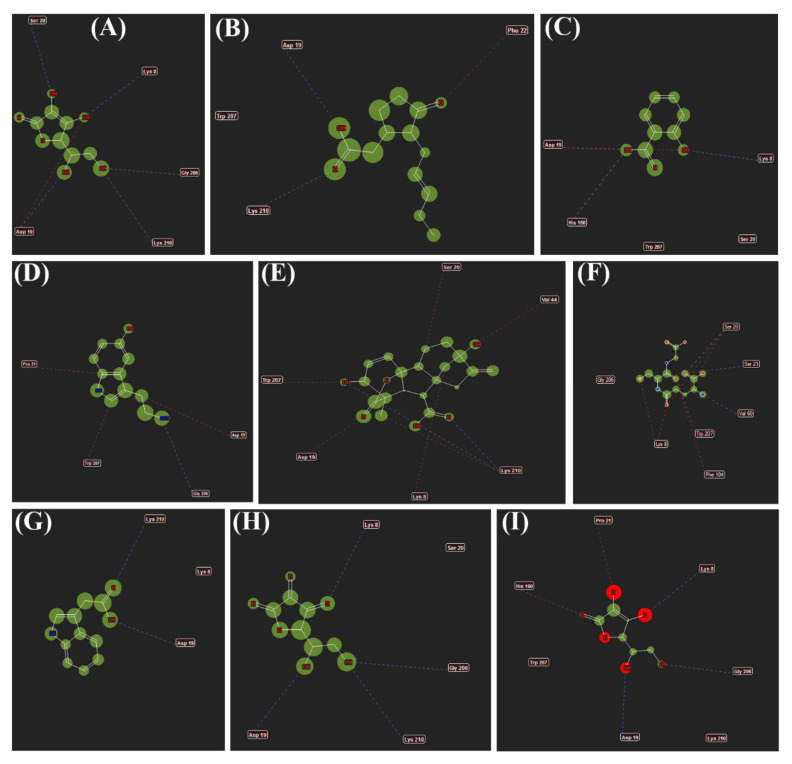
Interactions between the ligands and the active site of *Os*DHAR. (**A**) AsA; (**B**) JA; (**C**) SA; (**D**) 5-HT; (**E**) GA3; (**F**) GSH; (**G**) IAA; (**H**) DHA and (**I**) MDHA. The dotted lines represent hydrogen bonding and stearic interactions between the ligands and the amino acid residues shown.

Interactions between the ligands and the active site of *Os*DHAR. (A) AsA; (B) JA; (C) SA; (D) 5-HT; (E) GA3; (F) GSH; (G) IAA; (H) DHA and (I) MDHA are shown in Figure 6. The dotted lines represent hydrogen bonding and stearic interactions between the ligands and the amino acid residues shown.

**Figure 7:**
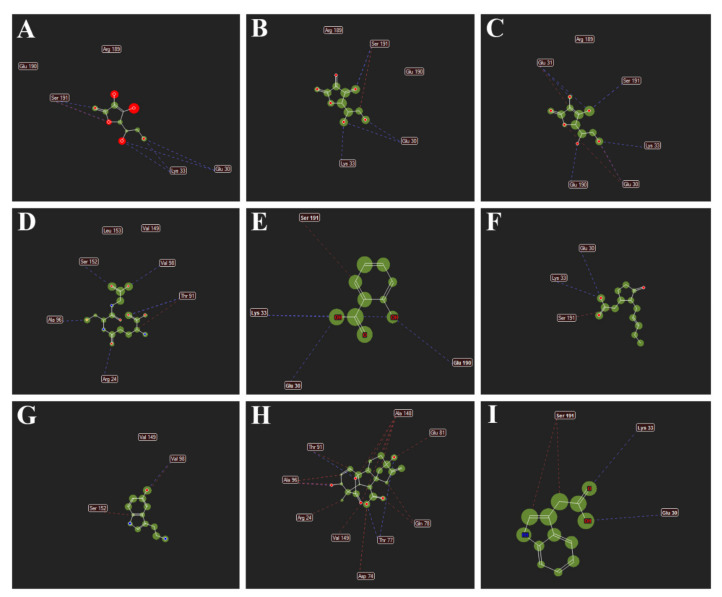
Interactions of different metabolites (**A**) MDHA; (**B**) DHA; (**C**) AsA; (**D**) GSH; (**E**) SA; (**F**) JA; (**G**) 5-HT; (**H**) GA3 and (**I**) IAA with *Ta*DHAR. The amino acids shown are the ones with which the metabolites form hydrogen bonding and stearic interactions.

Figure 7 represents the interactions of different metabolites such as (A) MDHA; (B) DHA; (C) AsA; (D) GSH; (E) SA; (F) JA; (G) 5-HT; (H) GA3 and (I) IAA with *Ta*DHAR. The amino acids shown are the ones with which the metabolites form hydrogen bonding and stearic interactions.

**Figure 8:**
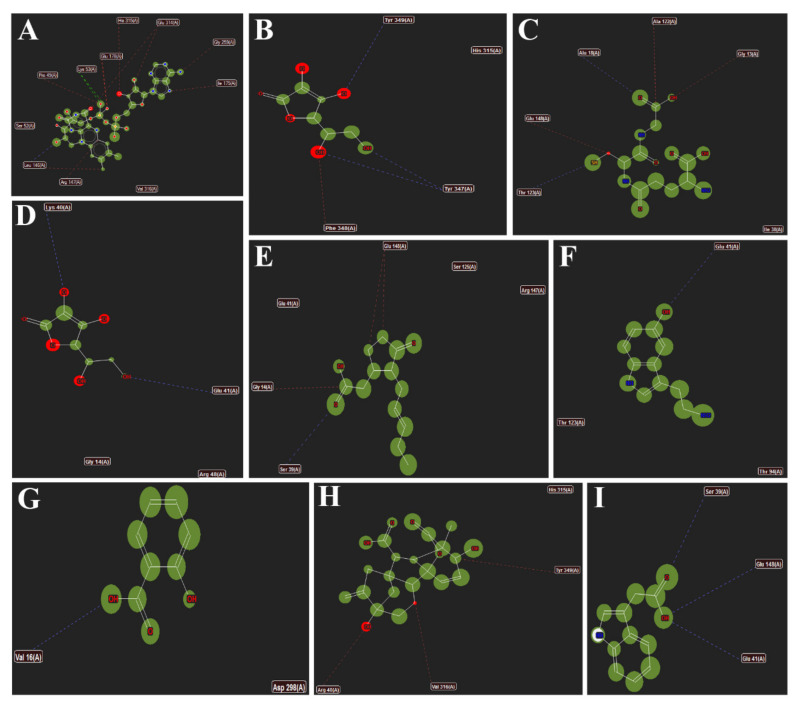
Interactions of different metabolites (**A**) Co-crystallized FAD; (**B**) MDHA; (**C**) GSH; (**D**) AsA; (**E**) JA; (**F**) 5-HT; (**G**) SA; (**H**) GA3 and (**I**) IAA with the active site amino acid resides of *Os*MDHAR. The amino acids shown are the ones with which the metabolites form hydrogen bonds and stearic interactions.

Figure 8 highlights the interactions of different metabolites (A) Co-crystallized FAD; (B) MDHA; (C) GSH; (D) AsA; (E) JA; (F) 5-HT; (G) SA; (H) GA3 and (I) IAA with the active site amino acid resides of *Os*MDHAR. The amino acids shown are the ones with which the metabolites form hydrogen bonds and stearic interactions. The interactions of *Zm*DHAR, *Zm*MDHAR and *Ta*MDHAR are provided in Figures S2–S4, respectively.

## 4. Discussion

In the present study, molecular docking was performed between different secondary metabolites and two metal ions (Al^3+^, Mn^2+^) with the DHAR and MDHAR of rice, maize and wheat. Here, the enzymes were taken as models, and the potential of the metabolites in conferring protection against the ions were analysed. When a metabolite binds to an enzyme, it may prevent interaction and/or binding of another. This depends on the affinities, which are measured as docking scores. Docking scores represent the amount of energy liberated when a compound docks/binds with an enzyme. A ligand having a higher docking (a more negative) score may potentially prevent another which has a lower (a less negative) docking score. The validity and accuracy of the present modelling study is revealed from the fact that co-crystallized ligands were found to bind to the same active site with the same orientation when docked using MVD. Additionally, the metal/metalloid ions showed no hydrogen bond scores (Table 2), which further validates the modelling study. Furthermore, compared to other similar modelling algorithms, MVD has a better accuracy [74]. It is known that several plant secondary metabolites, including AsA, GSH, GA3, JA, SA, 5-HT and IAA, confer protection against metal/metalloid-induced abiotic stresses [75,76,77,78,79,80,81,82,83,84,85,86,87,88]. The present study is an attempt to understand whether these metabolites may prevent the interactions of Al^3+^ and Mn^2+^ with the two critical anti-oxidant enzymes (DHAR and MDHAR) of rice, maize and wheat.

In the present study, it was found that the docking scores of the metal ions were lower compared to the metabolites in the case of all the targets (Table 2). This is largely because the ions provide lesser surfaces for interactions and binding, compared to the metabolites. However, among the five best docking poses of the ligands with the *Os*DHAR, both the ions bind at three different locations, and out of them only one of the poses overlaps partially with the electrostatic surfaces of the metabolites (Figure 2). In the case of *Ta*DHAR, while Al^3+^ binds at one, Mn^2+^ binds at two locations. The electrostatic surfaces of Al^3+^ and one of the poses of Mn^2+^ overlap with the surfaces of the metabolites (Figure 3). This indicates that the metabolites may potentially interfere with the interaction of the ions. However, no such overlapping occurs in the case of *Zm*DHAR (Figure S1), which indicates that the metabolites may not confer protection against these ions. In the case of *Os*MDHAR, both the metal ions bind at the active site of the enzyme at three locations, and out of them the surface of only one ion overlaps with all the metabolites, indicating a possible mode of protection (Figure 4). However, the other two MDHARs show no such interactions and overlaps (Figures S1, S3 and S4). Since the docking scores of the metal ions are lower than the secondary metabolites, and because they have overlapping electrostatic surfaces in the case of *Os*DHAR, *Ta*DHAR and *Os*MDHAR, it is argued that the metabolites may prevent the ions from interacting with the enzymes. Thus, the results demonstrate that the toxicity of the ions may be reduced or prevented in the presence of higher cellular quantities of the metabolites. Similar findings have recently been reported by Choudhury et al. [54] in rice using DHAR as a model. Thus, the present study hypothesizes a novel mechanism of protection by plant secondary metabolites against metal-induced abiotic stress. The study is novel as it hypothesizes a novel mechanism of amelioration of metal-induced abiotic stress by plant secondary metabolites in cereals.

However, the major limitation of the present study is that the structures of DHAR and MDHAR of maize and wheat were not available, and their three-dimensional models were obtained from the SWISS-MODEL database. This may be one of the factors which influenced the overlapping of the electrostatic surfaces. It is assumed that, with actual structures, a better modelling result may be obtained. Nevertheless, the findings are crucial in understanding the mechanism of action of plant metabolites in protecting metal-induced abiotic stress, oxidative stress in particular, in cereals.

## 5. Conclusions

Abiotic stresses, such as Al^3+^ and Mn^2+^ stress, affect the antioxidant defence mechanisms in plants and alters the cellular redox homeostasis. This not only affects growth and productivity, but maintaining the redox homeostasis remains a major challenge. The present study demonstrated that the stressors such as Al^3+^ and Mn^2+^ associated with acidic soils may potentially interact with the two critical antioxidant enzymes of the AsA-GSH cycle, DHAR and MDHAR, and possibly affect their activities. However, secondary metabolites, including AsA, GSH, JA, SA, IAA, GA3 and 5-HT may prevent interaction of the metal ions with *Os*DHAR, *Ta*DHAR and *Os*MDHAR. This is hypothesized to be affected by stronger interactions of the metabolites with the active sites of the enzymes. Because the findings hypothesize a novel mechanism in understanding the role of plant metabolites against metal-induced abiotic stress in cereals, it is highly significant. Thus, increasing the cellular levels of these metabolites through metabolic and genetic engineering, and plant breeding approaches, is hypothesized to ameliorate abiotic stress caused by the two metal ions.

## Figures and Tables

**Table 1:** Details of the compounds/ligands used in the study. XlogP3: Octanol-water partition coefficient; HBD: Number of hydrogen bond donor acceptor; HBA: Number of hydrogen bond acceptor; TPSA: Topological polar surface area.

Ligand	PubChem ID	Mol. Formula	Mol. Wt (g/mol)	XlogP3	HBD	HBA	TPSA (Å^2^)
Salicylic acid	338	C_7_H_6_O_3_	138.12	2.3	2	3	57.5
IAA	802	C_10_H_9_NO_2_	175.18	1.4	2	2	53.1
Serotonin	5202	C_10_H_12_N_2_O	176.21	0.2	3	2	62
GA3	6466	C_19_H_22_O_6_	346.4	0.2	3	6	104
GSH	124886	C_10_H_17_N_3_O_6_S	307.33	−4.5	6	8	160
DHA	440667	C_6_H_6_O_6_	174.11	−1	2	6	101
Jasmonic acid	5281166	C_12_H_18_O_3_	210.27	1.6	1	3	54.4
MDHA	53262277	C_6_H_7_O_6_	175.12	−1	2	5	90.8
Ascorbic acid	54670067	C_6_H_8_O_6_	176.12	−1.6	4	6	107
Aluminium ion	104727	Al^3+^	26.981538	-	0	0	0
Manganese ion	27854	Mn^2+^	54.93804	-	0	0	0

**Table 2:** Docking scores (MolDock, Rerank and Hydrogen bond) of DHAR of rice, maize and wheat with different ligands, including ions.

Ligands	*Os*DHAR			*Zm*DHAR			*Ta*DHAR		
	MolDock	Rerank	HBond	MolDock	Rerank	HBond	MolDock	Rerank	HBond
SA	−53.61	−42.70	−4.22	−65.25	−57.66	−8.19	−58.23	−52.20	−6.82
IAA	−87.50	−69.73	−5.15	−84.28	−60.29	−4.08	−80.75	−63.68	−4.90
5-HT	−72.01	−58.53	−4.99	−92.70	−50.79	−8.91	−79.74	−64.88	−2.50
GA3	−84.02	−70.38	−3.46	−99.20	−78.81	−5.72	−92.83	−58.92	−5.62
GSH	−91.60	−75.41	−5.74	−103.79	−85.35	−8.92	−96.60	−82.38	−9.31
JA	−93.23	−72.72	−4.60	−96.34	−18.03	−5.00	−84.62	−65.49	−4.62
AsA	−62.38	−56.41	−8.61	−72.86	−67.44	−9.82	−70.58	−63.03	−13.44
Al^3+^	−25.81	−30.59	0.00	−41.36	−34.43	0.00	−22.05	−21.93	0.00
Mn^2+^	−26.98	−30.41	0.00	−41.37	−34.42	0.00	−23.44	−21.66	0.00

**Table 3:** Docking scores (MolDock, Rerank and Hydrogen bond) of MDHAR of rice, maize and wheat with different ligands, including ions.

Ligands	*Os*MDHAR			*Zm*MDHAR			*Ta*MDHAR		
	MolDock	Rerank	HBond	MolDock	Rerank	HBond	MolDock	Rerank	HBond
SA	−69.24	−56.19	−5.00	−71.05	−59.15	−4.82	−70.20	−59.84	−5.78
IAA	−102.96	−83.96	−4.81	−100.38	−79.58	−5.73	−98.33	−81.03	−3.17
5-HT	−102.67	−84.13	−6.06	−99.78	−78.83	−4.12	−103.73	−84.33	−5.87
GA3	−116.56	−78.70	−6.36	−117.03	−60.76	−7.58	−119.83	−95.04	−5.81
GSH	−126.49	−78.36	−12.95	−125.39	−91.09	−15.93	−141.58	−122.03	−18.32
JA	−111.14	−91.62	−4.44	−109.56	−89.51	−7.06	−114.21	−92.53	−6.59
AsA	−81.00	−66.94	−8.07	−88.97	−80.97	−13.70	−89.68	−78.91	−12.48
Al^3+^	−41.24	−41.05	0.00	−39.50	−31.69	0.00	−32.45	−32.75	0.00
Mn^2+^	−41.24	−41.05	0.00	−39.51	−31.70	0.00	−32.45	−32.75	0.00

**Table 4:** Correlation analysis of docking scores of the DHAR of rice, maize and wheat with different ligand properties. NC: Not correlated.

Ligand Property	*Os*DHAR			*Zm*DHAR			*Ta*DHAR		
	MolDock	Rerank	Hbond	MolDock	Rerank	Hbond	MolDock	Rerank	Hbond
Mol. Wt	0.61	0.64	NC	NC	0.51	NC	NC	NC	NC
XlogP3	NC	NC	−0.51	NC	NC	−0.58	NC	NC	−0.58
HBD	NC	NC	0.60	NC	0.72	NC	NC	NC	NC
HBA	NC	NC	0.73	NC	NC	NC	NC	NC	0.68
TPSA	NC	NC	0.74	NC	0.53	NC	NC	NC	0.58

**Table 5:** Correlation analysis of docking scores (MolDock, Rerank and Hydrogen bond) of the MDHAR of rice, maize and wheat with different ligand properties. NC: Not correlated.

Ligand Property	*Os*MDHAR			*Zm*MDHAR			*Ta*MDHAR		
	MolDock	Rerank	Hbond	MolDock	Rerank	Hbond	MolDock	Rerank	Hbond
Mol. Wt	0.57	NC	NC	0.54	NC	NC	0.59	0.54	NC
XlogP3	NC	NC	−0.95	NC	NC	−0.87	NC	NC	−0.93
HBD	NC	NC	0.73	NC	NC	0.79	NC	NC	0.79
HBA	NC	NC	0.83	NC	NC	0.85	NC	NC	0.83
TPSA	NC	NC	0.90	NC	NC	0.88	NC	NC	0.89

## Data Availability

All the relevant data is available in the article and its Supplementary Materials.

## Supplementary Materials

The following supporting information can be downloaded at: https://www.mdpi.com/article/10.3390/antiox11030458/s1, Figure S1: Electrostatic surfaces of the metabolites and ions at ZmDHAR (A,B), ZmMDHAR (C,D) and TaMDHAR (E,F). In (A–D), surfaces of all the metabolites are shown in white, and Al^3+^ in yellow and Mn^2+^ in red. The figure shows that there is no overlap between the metabolites and ions; Figure S2: Interactions of different metabolites with the active site amino acid residues of ZmDHAR with (A) DHA; (B) GSH; (C) AsA; (D) JA; (E) 5-HT; (F) SA; (G) GA3; (H) IAA. The dotted lines represent hydrogen bonding and stearic interactions between the ligands and the amino acid residues shown; Figure S3: Interactions of different metabolites (A) MDHA; (B) DHA; (C) AsA; (D) GSH; (E) SA; (F) JA; (G) 5-HT; (H) GA3; (I) IAA with different amino acids of the active site of ZmMDHAR. The amino acids shown are the ones with which the metabolites form hydrogen bonds and stearic interactions; Figure S4: Interactions of the metabolites (A) MDHA; (B) DHA; (C) AsA; (D) GSH; (E) SA; (F) JA; (G) 5-HT; (H) GA3; (I) IAA with the active site residues of *Ta*MDHAR. The amino acids shown are the ones with which the metabolites form hydrogen bonds and stearic interactions.
