# Supplementary material for: A Computational Study of the Role of Secondary Metabolites for Mitigation of Acid Soil Stress in Cereals Using Dehydroascorbate and Mono-Dehydroascorbate Reductases
Source: Antioxidants (Basel). 2022 Feb 25;11(3):458. doi: 10.3390/antiox11030458 (PMC8944642; doi:10.3390/antiox11030458)
Supplement: Supplementary file 1 [file antioxidants-11-00458-s001.zip › antioxidants-1585380-supplementary.pdf]

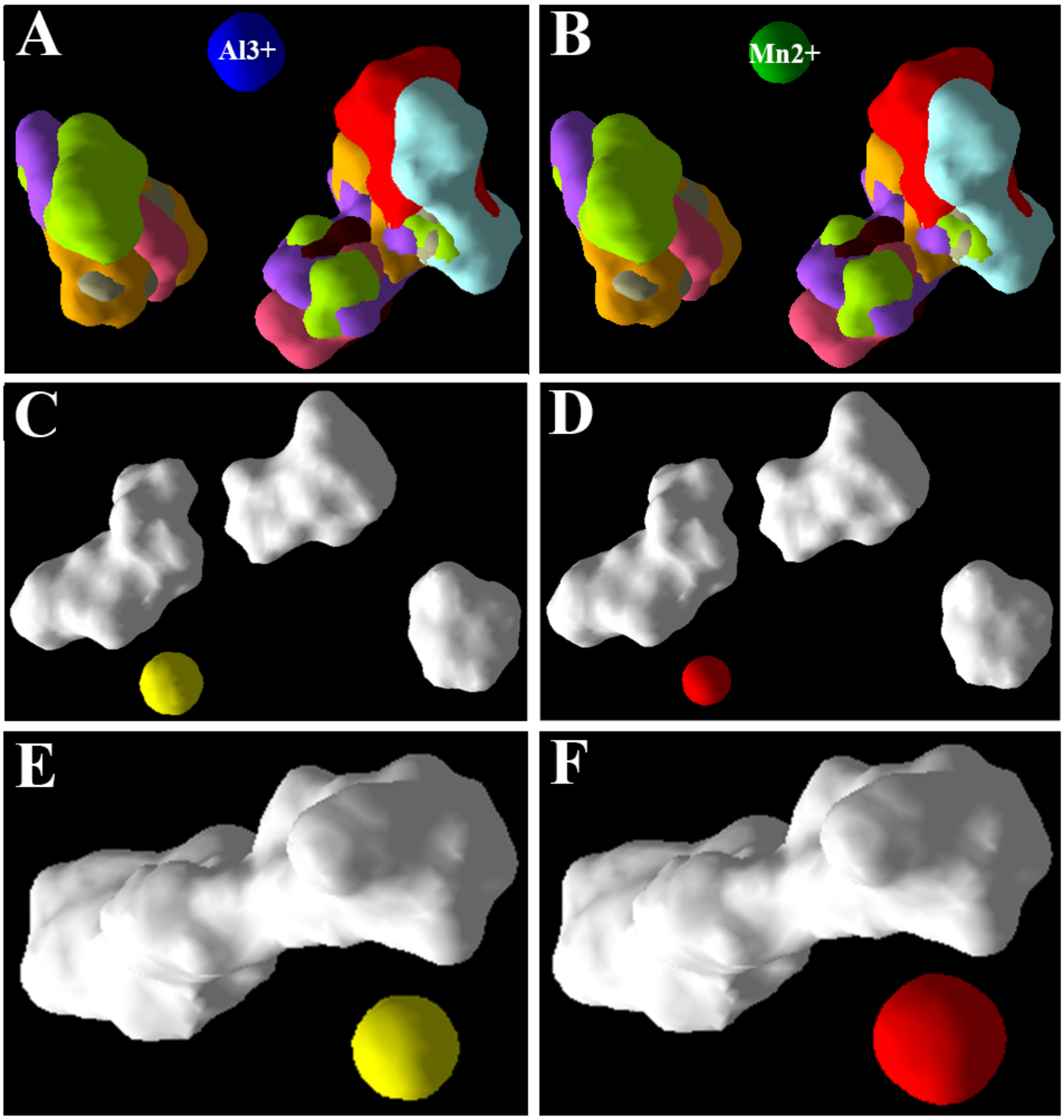

Figure S1: Electrostatic surfaces of the metabolites and ions at *Zm*DHAR (A, B), *Zm*MDHAR (C, D) and TaMDHAR (E, F). In A-D, surfaces of all the metabolites are shown in white, and Al^{3+} in yellow and Mn^{2+} in red. The figure shows that there is no overlap between the metabolites and ions.

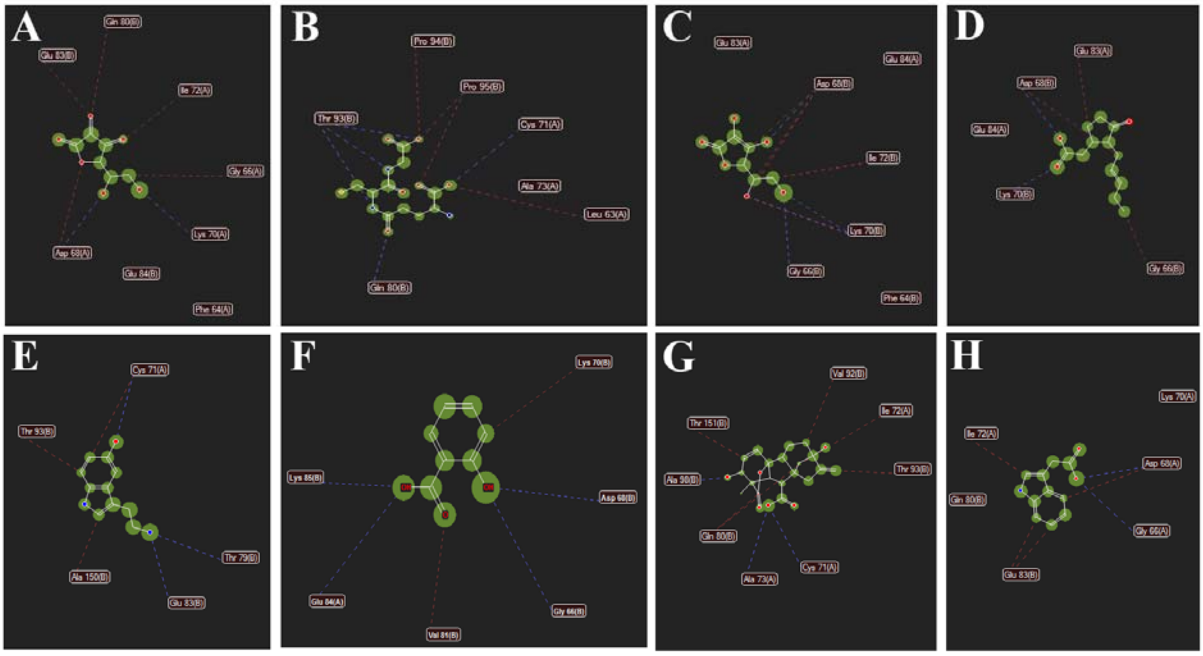

Figure S2: Interactions of different metabolites with the active site amino acid residues of *Zm*DHAR with (A) DHA; (B) GSH; (C) AsA; (D) JA; (E) 5-HT; (F) SA; (G) GA3; (H) IAA. The dotted lines represent hydrogen bonding and stearic interactions between the ligands and the amino acid residues shown.

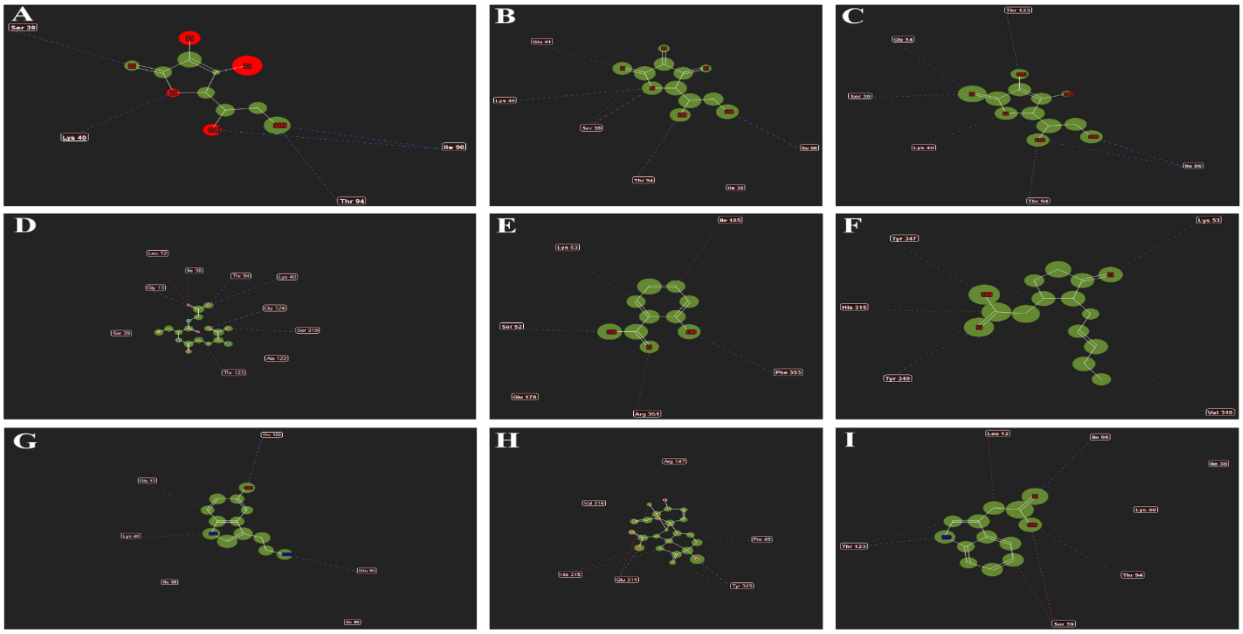

Figure S3: Interactions of different metabolites (A) MDHA; (B) DHA; (C) AsA; (D) GSH; (E) SA; (F) JA; (G) 5-HT; (H)GA3; (I) IAA with different amino acids of the active site of *Zm*MDHAR. Amino acids shown are the ones with which the metabolites form hydrogen bonds and stearic interactions.

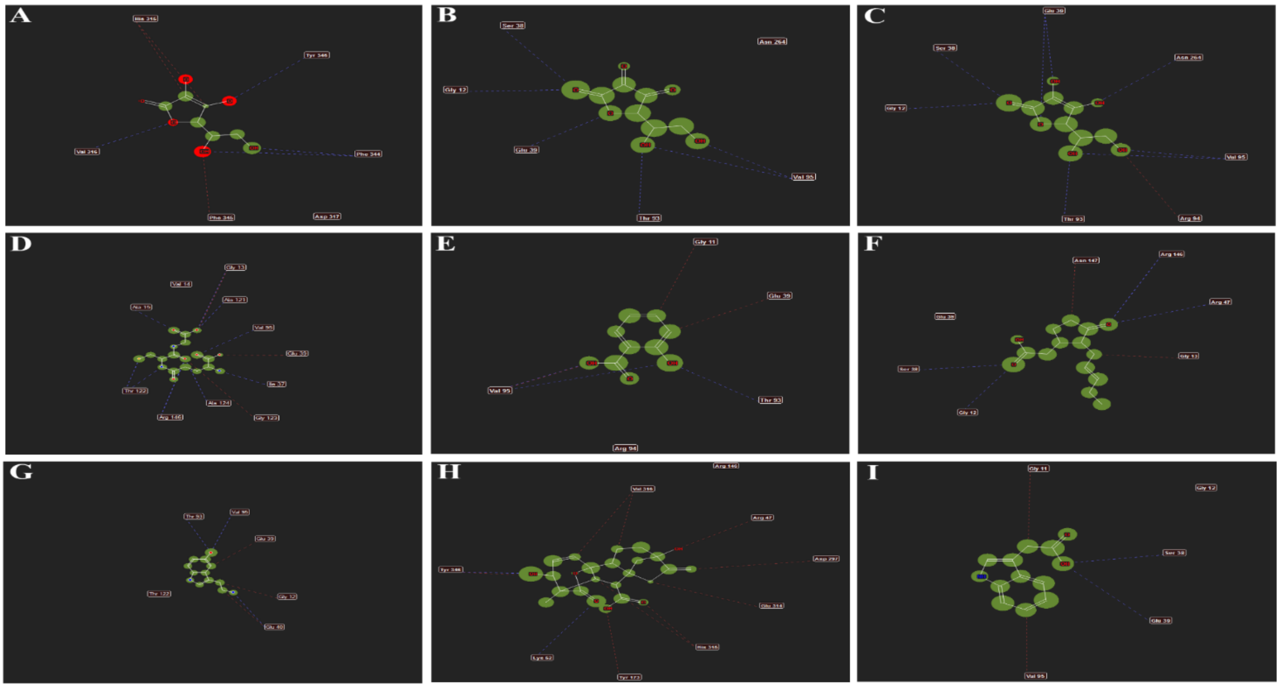

Figure S4: Interactions of the metabolites (A) MDHA; (B) DHA; (C) AsA; (D) GSH; (E) SA; (F) JA; (G) 5-HT; (H) GA3; (I) IAA with the active site residues of *Ta*MDHAR. Amino acids shown are the ones with which the metabolites form hydrogen bonds and stearic interactions.
